# Supplementary material for: Reconciling Mining with the Conservation of Cave Biodiversity: A Quantitative Baseline to Help Establish Conservation Priorities
Source: PLoS One. 2016 Dec 20;11(12):e0168348. doi: 10.1371/journal.pone.0168348 (PMC5173368; doi:10.1371/journal.pone.0168348)
Supplement: S1 Dataset — (ZIP) [file pone.0168348.s002.zip › Taxa/Serra Norte/SN_2013/N4WS/Lista N4WS-33.pdf]

CAVIDADE N4WS-0033

Classe	Ordem	Fam/Outros	Gên/Outros	Espécie	S-U	U-S	Zon.
Arachnida	Araneae	Drymusidae		jovem		X	E
Arachnida	Araneae	Filistatidae		sp.1	X	X	E
Arachnida	Araneae	Pholcidae	*Leptopholcus*	sp.1	X	X	E
Arachnida	Araneae	Pholcidae	*Mesabolivar*	*eberhard*	X		E
Arachnida	Araneae	Theraphosidae		sp.		1	E
Arachnida	Opiliones	Stygnidae	*Protimesius*	*gracilis*	1		E
Arachnida	Opiliones	Stygnidae	*Protimesius*	*laevis*	2		E
Arachnida	Pseudoscorpiones	Chernetidae	*Spelaeochernes*	sp.1		X	E
Entognatha	Collembola	Entomobryidae		sp.1	X		E
Insecta	Blattodea	Polyphagidae		jovem		2	E
Insecta	Diptera	Cecidomyiidae	Cecidomyiinae	sp.		X	E
Insecta	Diptera	Psychodidae	*Sciopemyia*	*sordellii*		X	E
Insecta	Hymenoptera	Formicidae	*Pachycondyla*	*striata*	1	1	E
Insecta	Isoptera	Termitidae	*Nasutitermes*	sp.	X	X	E
Insecta	Lepidoptera	Noctuoidea		sp.1		9	E
Insecta	Lepidoptera	Noctuoidea		sp.12		3	E
Insecta	Orthoptera	Phalangopsidae	*Paraclodes*	sp.		2	E
Insecta	Orthoptera	Phalangopsidae		sp.1		1	E
Insecta	Psocoptera	Psyllipsocidae		jovem	X		E
Amphibia	Anura	Strabomantidae	*Pristimantis*	cf. *fenestratus*	3	2	E
Ave	Caprimulgiformes	Caprimulgidae	*Nyctidromus*	*albicollis*		1	E
Mammalia	Chiroptera	Emballonuridae	*Peropteryx*	sp.	1		E
